# Single-cell transcriptomic analysis of the immune cell landscape in the aged mouse brain after ischemic stroke

**DOI:** 10.1186/s12974-022-02447-5

**Published:** 2022-04-07

**Authors:** Xuan Li, Jingjun Lyu, Ran Li, Vaibhav Jain, Yuntian Shen, Ángela del Águila, Ulrike Hoffmann, Huaxin Sheng, Wei Yang

**Affiliations:** 1 Multidisciplinary Brain Protection Program, Center for Perioperative Organ Protection, Department of Anesthesiology, Duke University Medical Center, P.O. Box 3094, Durham, NC 27710 USA; 2 Duke Molecular Physiology Institute, Duke University School of Medicine, Durham, NC USA

**Keywords:** Neuroinflammation, Transcriptome, scRNA-seq, Late reperfusion, Microglia, Neutrophil, Monocyte, Dendritic cell

## Abstract

**Background:**

Ischemic stroke is a medical emergency that primarily affects the elderly. A complex immune response in the post-stroke brain constitutes a key component of stroke pathophysiology. This study aimed to determine how stroke affects immune cell populations in the aged brain based on molecular profiles of individual cells.

**Methods:**

Single-cell RNA sequencing and a new transient ischemic stroke mouse model with late reperfusion were used.

**Results:**

We generated, for the first time, a composite picture of immune cell populations in the stroke aged brain at single-cell resolution. We discovered at least 6 microglial subsets in the stroke aged brain, including a potentially stroke-specific subtype. Moreover, we identified major cell subpopulations formed by infiltrated myeloid cells after stroke, and revealed their unique molecular profiles.

**Conclusions:**

This study provided the first scRNA-seq data set for immune cells in the stroke aged brain, and offered novel insights into post-stroke immune cell heterogeneity.

**Supplementary Information:**

The online version contains supplementary material available at 10.1186/s12974-022-02447-5.

## Introduction

Ischemic stroke is a leading cause of death and long-term disability that primarily affects the elderly. After ischemic stroke, acute cell death, release of damage-associated molecular patterns, and blood–brain barrier damage ensue. Consequently, resident microglia quickly respond by transitioning into various states, and circulating leukocytes—including neutrophils, monocytes, dendritic cells (DCs), and lymphocytes—infiltrate the brain, which together leads to a complex immune response in the stroke brain [1–4]. This profound immune activation critically contributes to stroke outcome, and has long been considered as a therapeutic target for stroke [4, 5]. To develop effective immunomodulatory therapies for stroke, a deep understanding of the major components of the stroke-induced immune response, such as individual immune cell populations, is required.

Indeed, as almost all immunomodulatory interventions that target immune cells in the stroke brain have failed in the clinic [6], it has been increasingly realized that we need a more nuanced understanding of the detrimental and beneficial roles of distinct immune subsets specifically in stroke. However, experimental stroke research on the immune cells in the brain has relied largely on prior knowledge (e.g., phenotypes and markers) of immune cell populations that have been identified and defined mostly by other fields. This approach is inherently inadequate not only for unbiased dissection of stroke-induced complex immune cell states, and but also for unmasking new stroke-specific immune cell subsets.

Notably, mounting single-cell transcriptomic studies have revealed remarkable immune diversity in the brain during aging and in certain brain disorders [7]. For example, the immune cell subsets in the brain specific to certain pathologic conditions, including Alzheimer’s disease and oxidative stress, have been identified using single-cell RNA sequencing (scRNA-seq) [8–11]. Only 2 scRNA-seq mouse studies in stroke have been recently reported [12, 13]. The study by Zheng et al. was not primarily focused on immune cells, and analyzed all brain cells on day 1 after stroke [12]. The other study targeted the late stroke phase (i.e., days 5 and 14 after stroke), and characterized immune cells with high CD45 expression (CD45^hi^) in the brain, which precluded analysis of most microglia with low CD45 expression (CD45^low^) [13]. In addition, both studies used young mice. Moreover, data have shown that infiltration of most immune cells into the brain may peak on post-stroke day 3 [2, 14]. Taken together, the immune cell landscape at the single cell level in aged brains during the acute phase remains critically to be defined. Thus, the present study aimed to perform de novo characterization of the transcriptional profiles of individual immune cells in the post-stroke aged brain using scRNA-seq. To further increase clinical relevance, we developed a new transient ischemic stroke model with late reperfusion.

## Methods

The method description is provided in Additional file 1: Methods, and the full gene names are listed in Additional file 1: Table S1.

## Results and discussion

### A new transient ischemic stroke model

Occlusion of middle cerebral artery (MCAO) is frequently found in ischemic stroke patients, and as such, transient MCAO models, particularly intraluminal filament MCAO models, are widely used in experimental stroke research [15]. Since in filament MCAO models, the whole MCA flow is blocked, large infarcts and high mortalities, even in young animals, are normally observed when a prolonged ischemic duration is applied. Thus, these mouse models typically use a relatively short MCAO period (i.e., 30–90 min) followed by reperfusion. However, most stroke patients are elderly and experience a longer ischemic episode. Thus, we first aimed to establish a transient ischemic stroke with late reperfusion model that is also applicable in aged mice.

**Fig. 1:**
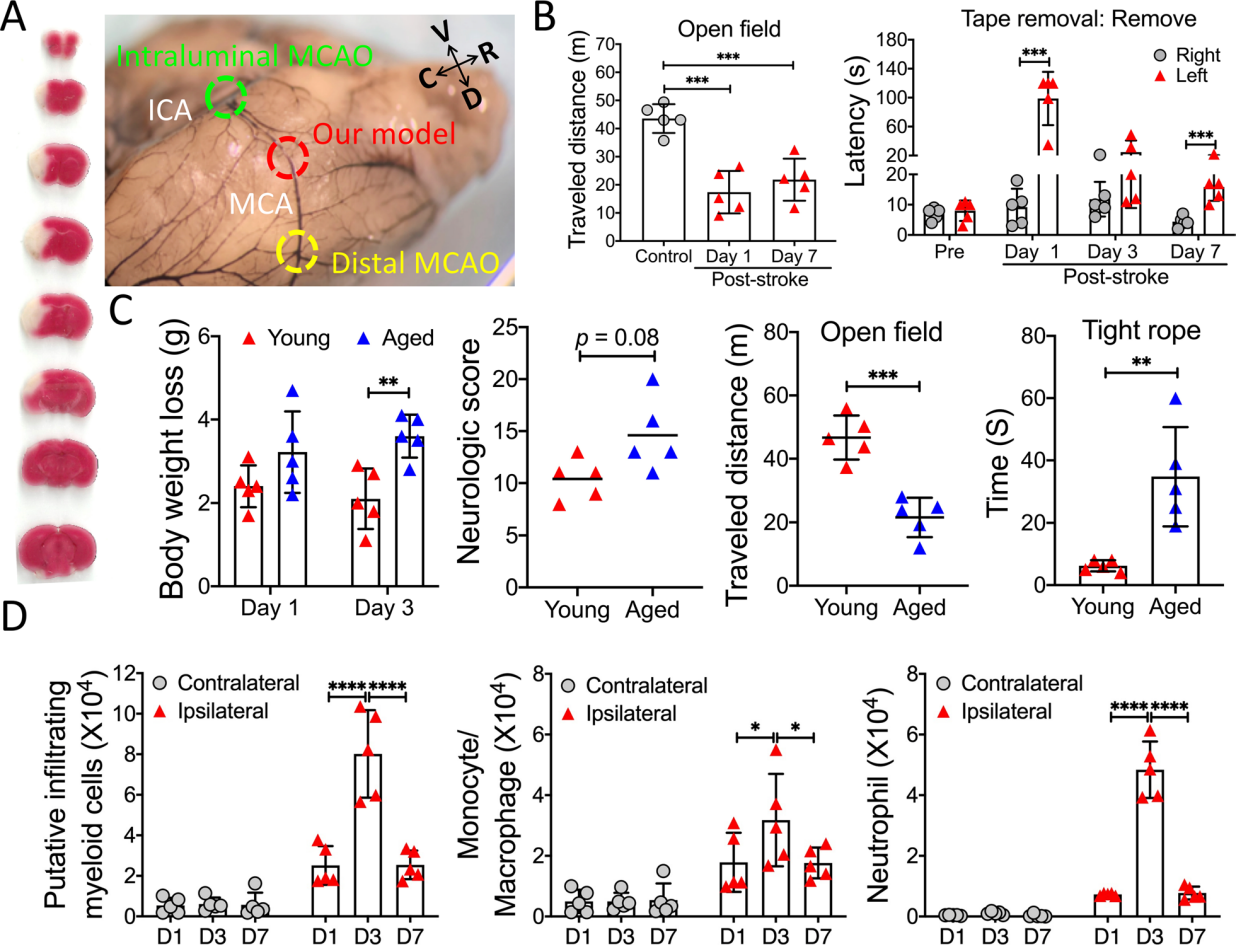
Modified transient transcranial MCAO (ttMCAO) mouse model.** A** Representative TTC stained brain (*left*) on day 3 after 6 h ttMCAO, and the MCA occlusion sites (*right*) of our model and existing models are shown. *V* ventral, *D* dorsal, *R* rostral, *C* caudal. **B** Functional outcome. Young mice were subjected to 6-h ttMCAO, and open field and tape removal tests were evaluated. Additional data from this experiment are shown in Additional file 1: Fig. S2. **C** Comparison of functional outcome between young and aged mice after ttMCAO. Young and aged mice were subjected to 6 h ttMCAO. All animals survived for 3 days. Body weight loss was evaluated on days 1 and 3 after stroke. On day 3 after stroke, mice were assessed by neurologic scoring, open field test, and tight rope test. **D** Time-course of immune profiling of infiltrating myeloid cells in the post-stroke young brain by flow cytometry. Young mice were subjected to 6-h ttMCAO, and immune cells in the brain were analyzed on post-stroke days 1(D1), 3 (D3), and 7 (D7). Data are presented as mean ± SEM or median (*n* = 5/group). *p < 0.05; **p < 0.01; ***p < 0.001; ****p < 0.0001

To prolong ischemic duration, one strategy is to occlude the MCA partially at a distal position through a small cranial window. A few distal MCAO mouse models, predominantly for permanent stroke, are available. They are rather similar and produce cortical infarcts, which results in excellent survival rates, but moderate functional deficits that can be difficult to detect. Taking all into account, we here developed a modified transient transcranial MCAO (ttMCAO) mouse model. The novelty of this new model lies mainly on the occlusion site and a long duration of ischemia (i.e., 6 h). In our ttMCAO model, we directly ligated the right MCA trunk proximal to the cortical branch to the rhinal cortex, which can cause brain damage in both cortex and striatum (Fig. 1A). This location is more proximal to the origin of the MCA branch that arises from the internal carotid artery (ICA), compared to those used in previous distal MCAO mouse models (Fig. 1A). To increase brain damage, the right common carotid artery (CCA) was also temporally ligated. After 6-h ischemia, both MCA and CCA were untied to initiate reperfusion. To determine whether reperfusion could be effectively achieved after such a long period of ischemia, we used laser speckle contrasting imaging (LSCI), ink-gelatin vascular visualization, and lectin-labeling of the vasculature (Additional file 1: Fig. S1). Data indicate that after removing sutures, the MCA-supplied region was evidently reperfused.

All young mice survived 6-h ttMCAO, and clearly showed neurologic deficits on post-stroke day 7 (Fig. 1B, Additional file 1: Fig. S2). Although aged mice also tolerated 6-h ttMCAO well, they exhibited significantly worse neurologic outcome vs young mice (Fig. 1C). Together, we have successfully established a new ischemic stroke model with late reperfusion.

Finally, we examined the dynamics of immune cell changes in the post-stroke brain by flow cytometry. Using a common gating strategy, cells with a high CD45 level (CD45^hi^) were classified as infiltrating leukocytes (Additional file 1: Fig. S3). We found that in young stroke mice, the increase in infiltrated leukocytes, especially monocytes/macrophages and neutrophiles (CD45^hi^CD11b^+^), started on post-stroke day 1, and peaked on day 3 (Fig. 1D), a timepoint that appears critical to studying stroke-induced immune cell changes [2]. Similar changes were observed in aged mice (Additional file 1: Fig. S4), although it seems that more infiltrated myeloid cells were found in aged vs young stroke brains.

### scRNA-seq analysis

We then set out to characterize immune populations in the stroke aged brain, and establish their molecular signatures based on unbiased transcriptional profiles by performing deep scRNA-seq analysis on all CD45^+^ cells collected from aged brains on day 3 after sham or ttMCAO.

**Fig. 2:**
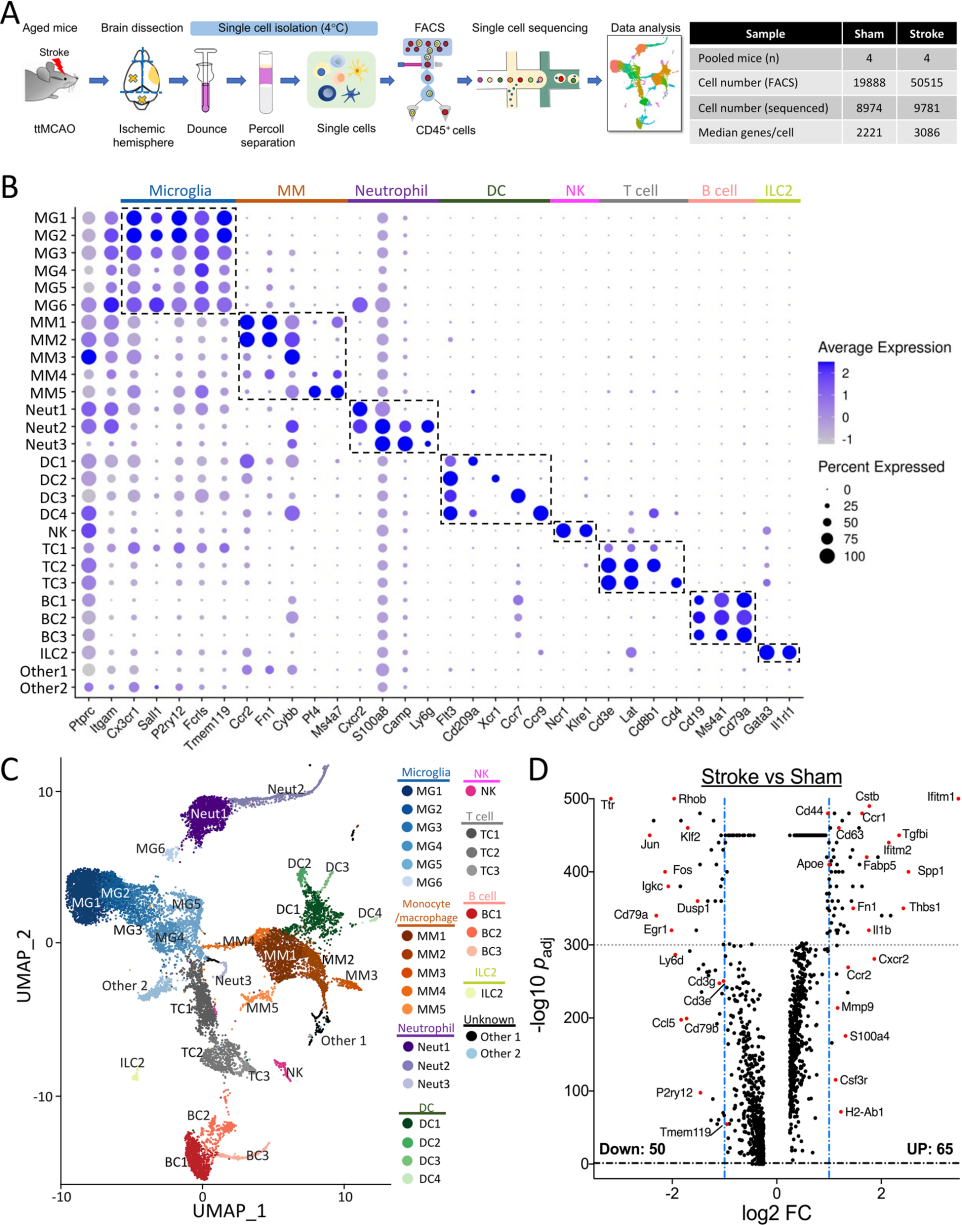
scRNA-seq analysis. **A** Workflow and data table of our scRNA-seq analysis. Aged mice were subjected to 6-h ttMCAO or sham. Three days later, both (sham) or ipsilesional (stroke) hemispheres were collected for scRNA-seq analysis. **B** Dot plot showing the scaled expression of selected signature genes for each cluster. Dot size depicts the percentage of cells within the cluster expressing each gene, and color intensity indicates the average expression level. **C** UMAP plot of aggregated data from both groups. **D** Volcano plot of differentially expressed genes (DEGs) between stroke and sham (Additional file 1: Table S4)

Obtaining high-quality cell samples is critical for scRNA-seq. Thus, we implemented the following components in our protocol (detailed in Additional file 1: Methods). First, to exclude most blood cells, mice were perfused with ice-cold Hank’s balanced salt solution. Second, to minimize transcriptional alterations, we avoided enzymes and used a mechanical dissociation protocol to prepare single-cell suspensions with all steps on ice or at 4 °C. Finally, to account for biological variations, we pooled cells from 4 mice for each sample. Single-cell suspensions were then stained with CD45-FITC and 7-AAD, and viable CD45^+^ cells were harvested by FACS for scRNA-seq analysis (Fig. 2A).

After rigorous quality control procedures, we performed unsupervised clustering of all sequencing data, which predicted 34 distinct clusters. We removed one contaminating cluster (no CD45 expression), and 5 clusters with small cell numbers. The remaining 28 clusters were then annotated into 8 major cell types (Fig. 2B, C; Additional file 1: Table S2), based on genes significantly overrepresented in each cluster [16]. Specifically, we identified 6 microglial (MG1–6), 5 monocyte/macrophage (MM1–5), 3 neutrophil (Neut1–3), 4 DC (DC1–4), 3 T-cell (TC1–3), and 3 B-cell (BC1–3) clusters, one nature killer (NK) cluster, one ILC2 (innate lymphoid cell 2) cluster, and 2 unknown clusters (Other1 and Other2). The top 50 differentially expressed gene (DEG) cluster markers are shown in Additional file 1: Table S3. We also identified potential marker genes for most clusters and projected them onto UMAP (Uniform Manifold Approximation and Projection) plots (Additional file 1: Fig. S5).

MG1–6 clusters expressed well-known microglial genes: *Cx3cr1, P2ry12, Fcrls, Sall1,* and *Tmem119*. MM1 and MM2 are the major monocyte/macrophage populations in the post-stroke brain, highly expressing *Ccr2, Fn1, and Cybb.* MM4 and MM5 are likely resident macrophages with high levels of *Pf4*, *Ms4a7*, and *Apoe*, and low *Ptprc* (CD45) [16]. MM3 cells may reflect infiltrated *Ccr2*^*−*^ monocytes, as they had the highest CD45 expression of all clusters. Among the 3 neutrophil subsets, the main Neut1 and Neut2 subsets exhibited high levels of CD45, *Itgam* (CD11b), and *Cxcr2*. All DC1–4 expressed the DC marker *Flt3*, but differed by signature expression of *CD209a* (DC1), *Xcr1* (DC2), *Ccr7* (DC3), and *Ccr9* (DC4). DC1 cells seem to be of monocyte origin with high *Ccr2* expression, while DC4 represents plasmacytoid DC (pDC), characterized by expression of *Ccr9, Cybb, Ly6c2,* and *Bst2.*

A minor NK cluster was reliably identified. All 3 T-cell subsets expressed the signature genes *Cd3e* and *Lat*. TC2 and TC3 cells can be designated as CD8^+^ and CD4^+^ T cells, respectively. TC1 cluster appears to be complex, which may reflect molecular changes after stroke. We identified 3 B cell subsets, all expressing *CD79a* and *Ms4a1*. A small number of ILC2 (an innate cell type) cells were also detected [17]. Interestingly, an increase of ILC2 cells in the stroke brain was recently reported in a preprint [18].

To determine the effects of stroke, we first analyzed aggregated data, similar to bulk RNA-seq analysis of all immune cells. We identified 115 DEGs (Fig. 2D; Additional file 1: Table S4). The up-regulated genes included *Mmp9*, *Il1b*, and *Thbs1*, consistent with previous reports. Some DEGs (e.g., *Cxcr2, Ccr2, Cd79a,* and *Cd3g*) reflected the increase or decrease in frequency of certain cell types after stroke. As shown below, such bulk analysis largely masked transcriptomic changes in individual cell subpopulations, highlighting the power of scRNA-seq in stroke research.

**Fig. 3:**
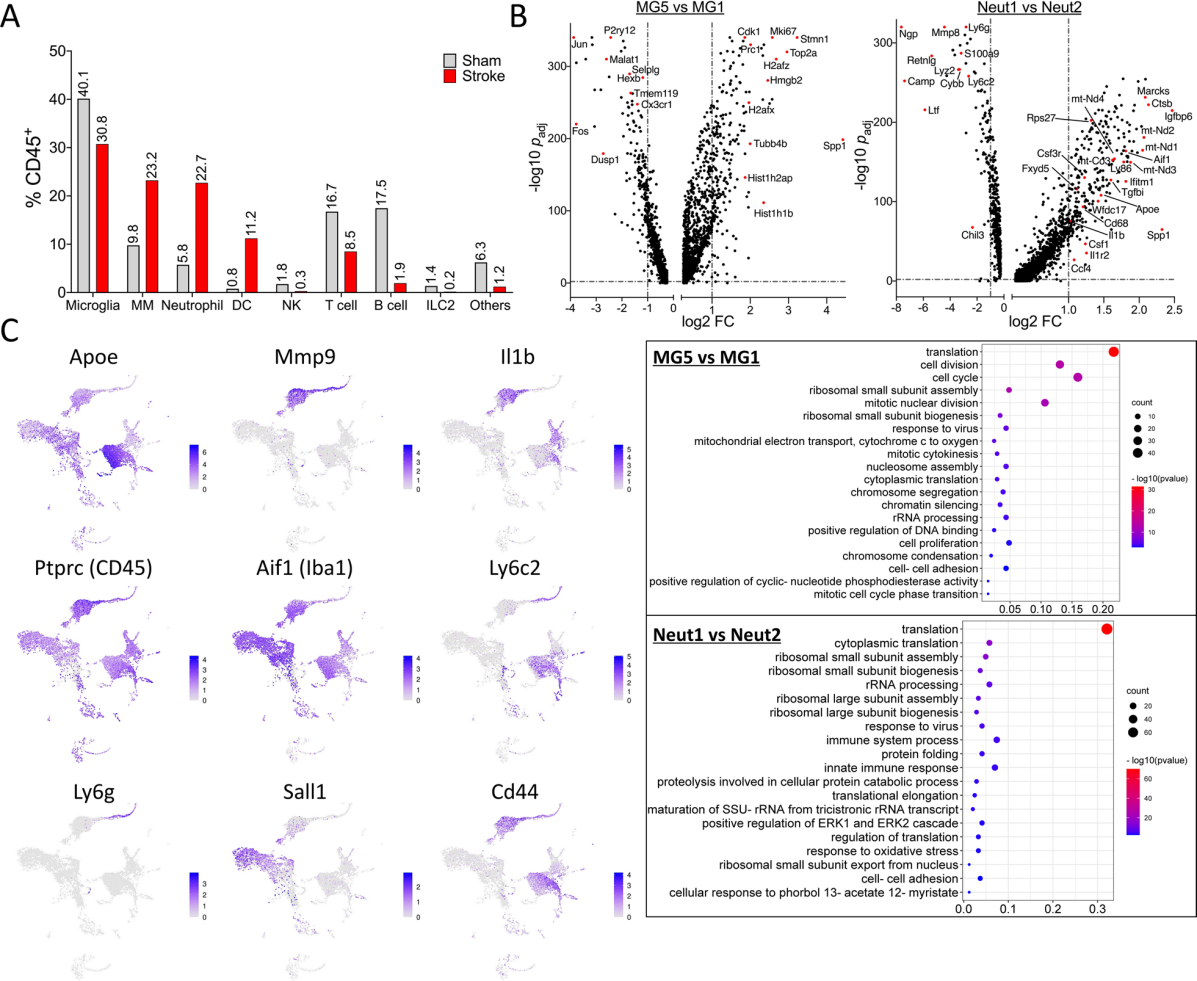
Immune landscape in the post-stroke aged brain revealed by scRNA-seq.** A** Changes in the frequency of major cell types in the aged brain on day 3 after stroke. **B** Volcano plots and the top 20 enriched gene ontology terms of biologic process for MG5 vs MG1 and Neut1 vs Neut2. **C** UMAP plots depicting expression of selected genes in the stroke aged brain

Overall, stroke profoundly altered the brain’s immune landscape (Fig. 3, Additional file 1: Fig. S6). In particular, the frequency of monocyte/macrophage, neutrophil, and DC cells was markedly increased (Fig. 3A). Below, we mainly focus on clusters with prominent frequency increases, i.e., MG5, MG6, MM1, Neut1, and DC1 (Additional file 1: Fig. S7).

Microglia, the most abundant immune cell type in the brain, are highly plastic [19]. However, their molecular heterogeneity in the post-stroke brain is incompletely understood. Notably, various microglial states with distinct molecular signatures have been proposed, e.g., disease-associated microglia [19]. In stroke research, microglia have often been classified using a simple M1 (pro-inflammatory)/M2 (anti-inflammatory) dichotomy, a likely oversimplified paradigm [20]. Our data uncovered at least 6 transcriptionally distinct microglial subsets in the stroke aged brain. MG1 and MG2 represent the resting state with high expression of homeostatic genes (e.g., *P2ry12* and *Tmem119*). After stroke, the frequency of MG1 and MG2 decreased, as these cells transitioned into different microglial subtypes, especially MG5, the predominant microglial subset in the stroke brain, and MG6. In MG5 cells, expression of *Top2A, Stmn1, Mki67,* and *Cdk1* was greatly up-regulated, indicating a highly proliferative state (Fig. 3B; Additional file 1: Table S5). On the other hand, homeostatic genes of microglia (e.g., *P2ry12, Tmem119, Cx3cr1,* and *Hexb*) were down-regulated in MG5, compared to resting MG1 (Fig. 3B). Interestingly, MG6 represents a distinctive microglial state present exclusively after stroke, and is in proximity to microglia and neutrophils on the UMAP plot (Fig. 2C). Indeed, besides microglial genes, MG6 cells expressed high levels of *Cxcr2, S100a8, Il1b, and Mmp9,* demonstrating a unique “neutrophil-like” phenotype (Additional file 1: Fig. S7D). This novel subtype may represent a stroke-specific state of microglia in the aged brain after stroke. Further functional characterization of MG6 subset may shed new light on the role of microglia in stroke pathophysiology.

The dominant monocyte/macrophage cluster in the post-stroke brain is MM1, increasing from 0.5% (sham) to 14.9% (stroke). Interestingly, MM1 cells characteristically express several potentially protective genes including *Apoe, Arg1, Ym1 (Chil3), Cd93, Hmox1, Tgfbi*, and *Hif1a,* while MM2 express *Cybb, Il1b, C3,* and *Hp* (Additional file 1: Table S3), suggesting that most monocyte/macrophages could be beneficial on post-stroke day 3.

Neut1 cluster likely formed from infiltrated neutrophils, increasing from 0.3% (sham) to 18.7% (stroke). In contrast to Neut2 cells that highly expressed the neutrophil homeostatic genes (e.g.*, ltf, Camp,* and *Ngp*)*,* Neut1 cells exhibited low expression of these genes, but upregulated expression of the mitochondrial membrane respiratory Complex I subunits (e.g., *mt-Nd1*–*4*), *Aif1*, *Ly86*, *Il1b*, and many protein synthesis-related genes, denoting a highly activated neutrophil state (Fig. 3B; Additional file 1: Table S6). Notably, Neut1 cells showed remarkably low mRNA expression of *Ly6g* (Figs. 2B, C), a commonly used neutrophil marker. This interesting observation may be not completely unexpected, according to a recent study by Grieshaber-Bouyer et al. [21]. In this study, the authors applied scRNA-seq to systemically analyze neutrophil populations in different mouse organs. They revealed a developmental spectrum from immature, mainly in bone marrow, to mature neutrophils, primarily in the blood and spleen. They also proposed a few gene markers to distinguish different developmental stages of neutrophils. For example, compared to pre-neutrophils, mature neutrophils exhibit lower expression of *Chil3*, *Camp*, *Ngp, Ltf, Lyz2, Cybb,* and *Ly6g,* and higher expression of *Wfdc17, Il1b, Ifitm1, Fxyd5, Rps27,* and *Csf3r*. Importantly, their dot plot data indicate an extremely low level of *Ly6g* mRNA in mature neutrophils. Thus, in light of their findings, Neut2 could be classified as immature neutrophils, while Neut1 represents infiltrating mature neutrophils from the blood after stroke (Fig. 3B).

DCs, the specialized antigen-presenting cells, have not been well-studied in stroke. Many peripheral DCs infiltrate the brain during the acute/subacute stroke phase [22, 23]. Consistently, we observed a massive increase of DCs in the post-stroke brain, especially DC1 cells. Notably, DC1 cells exhibited high expression of MHC class II-related genes, e.g., *H2-Aa, H2-Ab1* and *Cd74* (Additional file 1: Table S3), suggesting a potential contribution of DCs’ antigen-presenting function to stroke pathophysiology.

Finally, our analysis also revealed the following information (Fig. 3C). First, *Il1b, Mmp9, and Apoe* are critical to stroke pathogenesis; however, immune cell types expressing these genes after stroke remained elusive. Our data indicated that in the stroke aged brain, *Il1b* is largely expressed in neutrophils, DCs, and macrophages, but for microglia, only in MG6 subset; neutrophils and MG6 microglia appear to be major sources of *Mmp9*; and *Apoe* is primarily expressed in monocyte/macrophages. Second, *Aif1* (Iba1) has been described as a microglial marker. However, our data showed that A*if1* is also expressed in subsets of monocytes/macrophages, neutrophils, and DCs. Third, in line with previous reports [24, 25], *Sall1* and *Cd44* exhibited high specificity for microglia, and infiltrated myeloid cells, respectively. Finally, although CD11c (*Itgax*) is routinely used as a pan DC marker, our data indicated that CD11c is also found in NKs and monocytes/macrophages, and that *Flt3* could be an alternative DC marker in the aged stroke brain (Fig. 2B).

In summary, using a new transient ischemic stroke model with late reperfusion, we generated the first scRNA-seq data set for immune cells in the stroke aged brain, and provided novel insights into post-stroke immune cell heterogeneity in the brain. This study, therefore, constitutes an important step toward uncovering distinct immune subpopulations with unique functions in stroke pathophysiology.

## Supplementary Information

**Additional file 1.** Additional sections, figures and tables.

## Data Availability

All data generated or analyzed during this study are included in this published article and its Additional files.

## Abbreviations

CCA: Common carotid artery
DC: Dendritic cell
DEG: Differentially expressed gene
ICA: Internal carotid artery
ILC2: Innate lymphoid cell 2
LSCI: Laser speckle contrasting imaging
MCA: Middle cerebral artery
MCAO: MCA occlusion
NK: Nature killer
scRNA-seq: Single-cell RNA sequencing
ttMCAO: Transient transcranial MCAO
UMAP: Uniform manifold approximation and projection
