# Supplementary material for: Single-cell transcriptomic analysis of the immune cell landscape in the aged mouse brain after ischemic stroke
Source: J Neuroinflammation. 2022 Apr 7;19:83. doi: 10.1186/s12974-022-02447-5 (PMC8988369; doi:10.1186/s12974-022-02447-5)
Supplement: Supplementary file 1 — Additional file 1. Additional sections, figures and tables. [file 12974_2022_2447_MOESM1_ESM.zip › 12974_2022_2447_MOESM1_ESM/Addtional file 1_ESM.pdf]

Additional file 1

Single-cell transcriptomic analysis of the immune cell landscape in the aged mouse brain after ischemic stroke

Xuan Li, MD[1]; Jingjun Lyu, MD[1]; Ran Li, MD[1]; Vaibhav Jain, PhD[2]; Yuntian Shen, PhD[1]; Ángela del Águila, PhD[1]; Ulrike Hoffmann, MD[1]; Huaxin Sheng, MD[1]; Wei Yang, PhD[1]

[1] Multidisciplinary Brain Protection Program, Center for Perioperative Organ Protection, Department of Anesthesiology, Duke University Medical Center, Durham, NC, USA

[2] Duke Molecular Physiology Institute, Duke University School of Medicine, Durham, NC, USA

Methods

Animals. Animal experiments were approved by the Duke University Medical Center Animal Care and Use Committee. All studies were conducted in accordance with the United States Public Health Service's Policy on Humane Care and Use of Laboratory Animals. Young adult C57Bl/6 mice (3-4 months old) were purchased from The Jackson Laboratory (Maine). Aged C57Bl/6 mice (19-20 months old) were obtained through the NIA Aged Rodent Colonies program. Since this study was not focused on pre-clinical outcome but on the general immune response in aged mice, only male mice were used. The online tool Quickcalcs was used to randomize animals for group assignments. Due to obvious differences between young vs aged mice and sham vs stroke mice, blinding was not feasible.

Stroke surgery. We aimed to establish a transient stroke model that applies a relatively long ischemic period, has a good survival rate, produces readily detectable neurologic deficits, and can be used in aged animals. To this end, we developed a modified transient transcranial middle cerebral artery occlusion (ttMCAO) model, based on our previous permanent ischemic stroke model.[1] Briefly, mice were anesthetized with isoflurane, orally intubated, and mechanically ventilated. Rectal temperature was maintained at 37°C ± 0.2°C throughout the procedure. After a small neck midline incision was made, the right common carotid artery (CCA) was isolated and ligated using a 4-0 silk suture. A small transverse incision was then made between the eye and ear. Next, the lowest part of the temporal muscle was cut using a high temperature loop, the tip of the forceps was used to slightly push the temporal muscle aside to reveal the zygomatic arch, and a 3-mm segment of the zygomatic arch was removed. A small window (1-2 mm^2) was then drilled in the skull above the MCA. The MCA trunk was lifted with an 8-0 needle, and ligated with a 4-0 silk suture proximal to the cortical branch to the rhinal cortex (Fig. 1A) to initiate ischemia. Anesthesia was then terminated, and mice were allowed to recover and extubated. At the end of 6 hours ischemia, mice were anesthetized and intubated again. The sutures around the MCA and CCA were untied and removed. During model development, we used laser speckle contrast imaging (LSCI) to monitor changes in cerebral blood flow (CBF) flux before and during occlusion of the MCA, and after

reperfusion.

Assessment of reperfusion status. To examine reperfusion in our new 6-hour ttMCAO stroke model, we used the following 3 approaches.

Laser speckle contrast imaging (LSCI). LSCI was performed using a full-field laser perfusion imager RFLS (RWD Life Science Co, Kent, Delaware, USA), as described previously.[2] In addition to stroke surgery, a midline scalp incision was made to expose the skull. The same imaging parameters were used as before.[2]

Ink perfusion. Brain vessels were visualized using a gelatin-India ink casting method, as described previously.[3] Briefly, mice were deeply anesthetized with isoflurane, and transcardially perfused with 15 mL saline, followed by 15 mL 10% formalin. Next, 20 mL of the gelatin-India ink mixture (pre-heated to 40°C) was infused into the mice with the perfusion pressure controlled at 100 mmHg. Immediately, mice were covered with ice for at least 1 hour to allow gelatin solidification. Finally, brains were harvested and immersed in 10% neutral buffered formalin. Blood vessels were imaged with a Leica microscope.

Lectin labeling. Under isoflurane anesthesia, mice were injected with 100 µL saline solution containing 50 µg of DyLight- 594 lectin (Vector Laboratories) via the jugular vein. After 15 minutes, mice were transcardially perfused with saline, and then fixed with 4% paraformaldehyde. Mouse brains were collected, immersed in 30% sucrose in PBS for 2 days, and then stored at -80°C. Frozen brain sections (25 µm thick) were obtained using a Leica cryostat. Images were captured on a Zeiss Axio Imager Z2 motorized fluorescent microscope (Carl Zeiss MicroImaging).

Behavioral tests. The following functional assessments were performed to evaluate functional deficits after stroke, essentially as described previously.[4, 5]

Open field. Mice were placed in the center of an open field chamber (50 × 50 × 50 cm). Spontaneous locomotor activity was recorded for 10 minutes, and the video data were

analyzed by Topscan 3.0 software (CleverSys).

Tape removal. The tape removal test was used to evaluate sensory-motor impairments after stroke. Before surgery, mice were trained with one trial per day for 4 days. During the test, a small adhesive patch (0.3 cm × 0.4 cm) was applied to each forepaw. Each mouse was then placed in a transparent box, and observed for 2 minutes. The time lapse to contact and remove each adhesive tape was recorded.

Tight rope. Each mouse was placed with its forepaws at the middle of the rope (60 cm long), and the time lapse for the mouse to reach the platform was recorded. Each test had 2 trials, and the average time was calculated.

Neurologic scoring. A 48-point scoring system was used.[6] This scoring system evaluates general status (spontaneous activity, body symmetry, gait; 0-12), simple motor deficit (forelimb asymmetry, circling, hind-limb placement; 0-14), complex motor deficit (vertical screen climbing, beam walking; 0-8), and sensory deficit (hind limb, trunk, vibrissae, and face touch; 0-14). The final score was the sum of the 4 individual scores, with 0 = no deficit and 48 = maximal deficit.

Flow cytometry analysis. Our standard protocol was used.[7] Briefly, mice were deeply anesthetized with isoflurane, and transcardially perfused with ice-cold PBS. Brains were rapidly excised, and split into ipsilateral and contralateral hemispheres along the midline. Brain tissues were then cut into small pieces with scissors in ice-cold DMEM medium, followed by enzymatic digestion with Collagenase D (1 mg/mL) and DNAse I (0.1 mg/mL) (Sigma-Aldrich, St. Louis, MO, USA) for 45 minutes at 37°C. Cells were then filtered through a 70-μm cell strainer, and resuspended in a 70%/37% Percoll gradient (GE Healthcare Life Sciences, Pittsburgh, PA, USA). After centrifugation at 500 x g without braking, the immune cells at the interphase were harvested. After washing with 25 mL PBS, cell pellets were resuspended in 200 μL RPMI 1640 containing 2% fetal calf serum. Cell numbers were counted with a hemocytometer after staining with trypan blue. After incubation with Fc receptor blocking solution for 15 minutes, cells were immunostained

with combinations of different surface antibodies. The antibodies (all from BioLegend) included: CD45-FITC (103108), CD11b-PE/cy7 (101216), Ly6G-APC/cy7 (127624). LIVE/DEAD fixable violet kit (L34963A; Invitrogen) was used to stain dead cells. Flow cytometry data were acquired on FACS Canto (BD Biosciences, San Jose, CA, USA) and analyzed using FlowJo software.

Single-cell RNA sequencing (scRNA-seq) analysis. The scRNA-seq analysis was performed at Duke Molecular Genomics Core (MGC) following established protocols.

Preparation of single-cell suspension samples. We adopted a published protocol with modifications.[8] To minimize changes in gene expression during the procedure, each step was performed on ice or at 4°C, whenever possible. Mice were transcardially perfused with ice-cold Hank's balanced salt solution (HBSS), and the brains were quickly excised. The ipsilateral hemisphere of stroke brains and both hemispheres of sham brains were used for sample preparation. Brain tissues were cut into small pieces, and then homogenized with a 2-mL Dounce homogenizer (Kimble) in ice-cold HBSS using the loose and then tight pestles with 15-20 strokes each. Next, cells were filtered through a 70-µm cell strainer, resuspended in a 70%/37% Percoll gradient, and centrifuged to obtain the enriched immune cells at the interphase. The collected interphase cells were washed with 10 mL ice cold HBSS, and resuspended in 200 µL ice cold FACS buffer (0.5% BSA, 1 mM EDTA, in PBS) containing anti-CD45-FITC (103108; BioLegend) and 7-AAD (420404; BioLegend) for 30 minutes at 4°C. Cell samples were then washed in ice cold FACS buffer, centrifuged for 5 minutes at 300 x g, and resuspended in 500 µL ice cold FACS buffer.

Fluorescence-activated cell sorting (FACS). Cells were sorted on a BD FACSAria II with a purity mode. After gating live/dead cells, all $CD45^+$ live cells were collected into tubes. FACS-isolated cells were immediately delivered to Duke Molecular Genomics Core.

Library preparation. Cell suspensions were loaded on a 10x Genomics Chromium Controller Single-Cell Instrument (10x Genomics, Pleasanton, CA, USA), and mixed with

reverse transcription (RT) reagents along with gel beads and oil to generate single-cell gel beads in emulsions (GEMs). GEM-RT was performed in an Eppendorf Mastercycler Pro machine using the following cycle: 53°C for 45 minutes, 85°C for 5 minutes; held at 4°C. After RT, GEMs were broken, and the single-strand cDNA was purified with DynaBeads MyOne Silane Beads (Thermo Fisher Scientific). The cDNA was then amplified using the following protocol: 98°C for 3 minutes; cycled 11-13 ×: 98°C for 15 seconds, 67°C for 20 seconds, and 72°C for 1 minute; 72°C for 1 minute; held at 4°C. The amplified cDNA product was purified using the SPRIselect Reagent Kit (0.6 × SPRI) (Beckman Coulter). Indexed sequencing libraries were constructed using the reagents in the Chromium Single-Cell 3′ v3.1 Library Kit, following these steps: (1) fragmentation, end repair, and A-tailing; (2) SPRIselect cleanup; (3) adapter ligation; (4) postligation cleanup with SPRIselect; (5) sample index PCR; and (6) PostindexPCR cleanup. The barcoded sequencing libraries were quantified by quantitative PCR (KAPA Biosystems Library Quantification Kit for Illumina platforms). Sequencing libraries were transferred to the Duke Genomic and Computational Biology (GCB) Core, and loaded on a Novaseq 6000 (Illumina, San Diego, CA, USA) for sequencing.

Bioinformatics processing and data analysis. The primary analytical pipeline for the scRNA-seq analysis followed the recommended protocols from 10X Genomics. Briefly, we demultiplexed raw base call (BCL) files generated by Illumina sequencers into FASTQ files, upon which alignment to mm10-3.0.0 Mouse transcriptome, filtering, barcode counting, and UMI counting were performed using 10X's Cell Ranger software version 5.0.0. The secondary statistical analysis was performed using an R package Seurat version 4.0 which performed quality control and subsequent analyses on the feature-barcode matrices produced by Cell Ranger. In Seurat, data were first filtered for minimum gene (200) and cell observance frequency cut-offs (10) for both samples. We then closely examined the data and performed further filtering based a range of metrics (% mitochondrial expression < 20 for both samples, 250 < nFeature > 7500, 500 < nUMI(count) >50,000 for sham & 250 < nFeature >8000, 500<nUMI >55,000 for stroke) to identify and exclude possible multiplets (ie, instances where more than one cell was present and sequenced in a single emulsified gel bead) followed by normalization and

scaling samples. To further remove doublets/multiplets, DoubletFinder (version 2.0.3) was used in R individually on each sample.[9] The removal of further technical artifacts was performed using regression methods to reduce noise.

After quality control procedures, we performed linear dimensional reduction calculating principal components using the most variably expressed genes in our dataset. Library size and/or the numbers of genes expressed across subsets of cells may necessitate the restriction of cells upon which the variably-expressed genes are selected for inclusion when calculating principal components.[10] Significant principal components for downstream analyses were determined through methods mirroring those implemented by Macosko et al,[10] and these principal components (64) were carried forward for two main purposes: to perform cell clustering and to enhance visualization.[11] Cells were grouped into an optimal number of clusters for de novo cell type discovery using Seurat's FindNeighbors() and FindClusters() functions, graph-based clustering approaches with visualization of cells was achieved through the use of manifold learning technique UMAP (Uniform Manifold Approximation and Projection), which reduces the information captured in the selected significant principal components to 2 dimensions.[11, 12]

Differential expression of relevant cell marker genes was visualized on UMAP plot to reveal specific individual cell types. Additional downstream analyses included examining the cellular distribution of a priori genes of interest, closer examination of genes associated with cell clusters, and the refined clustering of cells in order to identify further resolution of cell types, in addition to comparing differences between experiments of different states. Combining multiple libraries using the integration strategy described in Stuart and Butler et al[13] allowed for calculation of differential expression, not only between clusters, but also within clusters across libraries using default parameters in Seurat. This allowed calculation of differential expression within cell type between sham and stroke brains. Enrichment analysis of gene ontology categories was performed using DAVID online tools (https://david.ncifc rf.gov). For all volcano plots (Fig. 2D and Fig. 3B), if a p_{adj} equals zero, the value of $-\log_{10}p_{adj}$ is randomly assigned a number between 300-500 in order to be plotted.

Statistical Analysis. The group size for each experiment was based on our previous studies or pilot experiments. Statistical analyses were performed using unpaired Student's *t*-test for 2 group comparisons, and one-way or two-way ANOVA with *post hoc* Holm-Sidak correction for multiple comparisons for more than 2 groups. Neurologic scores were analyzed with the Mann-Whitney *U* test. Data are presented as mean ± SEM or median. The level of significance was set at $p < 0.05$.

Table S1. List of full gene names.

Gene symbol	Gene name
Aif1(Iba1)	allograft inflammatory factor 1 (induction of brown adipocytes 1)
Apoe	apolipoprotein E
Arg1	arginase 1
Bst2	bone marrow stromal cell antigen 2
C3	complement component 3
Camp	cathelicidin antimicrobial peptide
Ccr2	chemokine (C-C motif) receptor 2
Ccr7	chemokine (C-C motif) receptor 7
Ccr9	chemokine (C-C motif) receptor 9
Cdk1	cyclin-dependent kinase 1
Cd19	CD19 antigen
Cd209a	CD209a antigen
Cd4	CD4 antigen
Cd44	CD44 antigen
Cd74	CD74 antigen
Cd79a	CD79A antigen
Cd8b1	CD8 antigen, beta chain 1
Cd93	CD93 antigen
Cd3e	CD3 antigen, epsilon polypeptide
Cd3g	CD3 antigen, gamma polypeptide
Chil3	Chitinase-like 3
Csf3r	colony stimulating factor 3 receptor
Cx3cr1	chemokine (C-X3-C motif) receptor 1
Cxcr2	chemokine (C-X-C motif) receptor 2
Cybb	cytochrome b-245, beta polypeptide
Fcrls	Fc receptor-like S, scavenger receptor
Flt3	FMS-like tyrosine kinase 3
Fn1	fibronectin 1
Fxyd5	FXYD domain-containing ion transport regulator 5
Gata3	GATA binding protein 3
H2-Aa	histocompatibility 2, class II antigen A, alpha
H2-Ab1	histocompatibility 2, class II antigen A, beta 1
Hexb	hexosaminidase B
Hif1a	hypoxia inducible factor 1, alpha subunit
Hmox1	heme oxygenase 1
Hp	haptoglobin
Ifitm1	interferon induced transmembrane protein 1
Il1b	interleukin 1 beta
Il1rl1	interleukin 1 receptor-like 1
Itgam	integrin alpha M
Itgax (CD11c)	integrin alpha X
ltf	lactotransferrin
Klre1	killer cell lectin-like receptor family E member 1
Lat	linker for activation of T cells

Ly6c2	lymphocyte antigen 6 complex, locus C2
Ly6g	lymphocyte antigen 6 complex, locus G
Ly86	lymphocyte antigen 86
Lyz2	lysozyme 2
mt-Nd1-4	mitochondrially encoded NADH dehydrogenase 1-4
Mki67	antigen identified by monoclonal antibody Ki 67
Mmp9	matrix metallopeptidase 9
Ms4a1	membrane-spanning 4-domains, subfamily A, member 1
Ms4a7	membrane-spanning 4-domains, subfamily A, member 7
Ngp	neutrophilic granule protein
Ncr1	natural cytotoxicity triggering receptor 1
Rps27	ribosomal protein S27
P2ry12	purinergic receptor P2Y, G-protein coupled 12
Pf4	platelet factor 4
Ptprc (CD45)	protein tyrosine phosphatase, receptor type, C
S100a8	S100 calcium binding protein A8 (calgranulin A)
S100a9	S100 calcium binding protein A9 (calgranulin B)
Sall1	spalt like transcription factor 1
Stmn1	stathmin 1
Thbs1	thrombospondin 1
Tmem119	transmembrane protein 119
Tgfbi	transforming growth factor, beta induced
Top2A	topoisomerase (DNA) II alpha
Wfdc17	WAP four-disulfide core domain 17
Xcr1	chemokine (C motif) receptor 1
Ym1(Chil3)	chitinase-like 3

Table S2 (Excel file).
Description: Average expression level of detected genes for each cluster.

Table S3 (Excel file).
Description: This file has 28 worksheet tabs. Each tab contains the top 50 genes enriched in the corresponding cluster. These genes were used to identify cell types.

Table S4 (Excel file).
Description: The list of differentially expressed genes (DEGs) between stroke and sham. The analysis was performed on aggregated data from all clusters. The selection criteria are: p_{adj} < 0.01 and fold change >= 2 or =< -2.

Table S5 (Excel file).
Description: The list of differentially expressed genes (DEGs) between cluster MG5 and MG1. The selection criteria are: p_{adj} < 0.01 and fold change >= 2 or =< -2.

Table S6 (Excel file).
Description: The list of differentially expressed genes (DEGs) between cluster Neut1 and Neut2. The selection criteria are: p_{adj} < 0.01 and fold change >= 2 or =< -2.

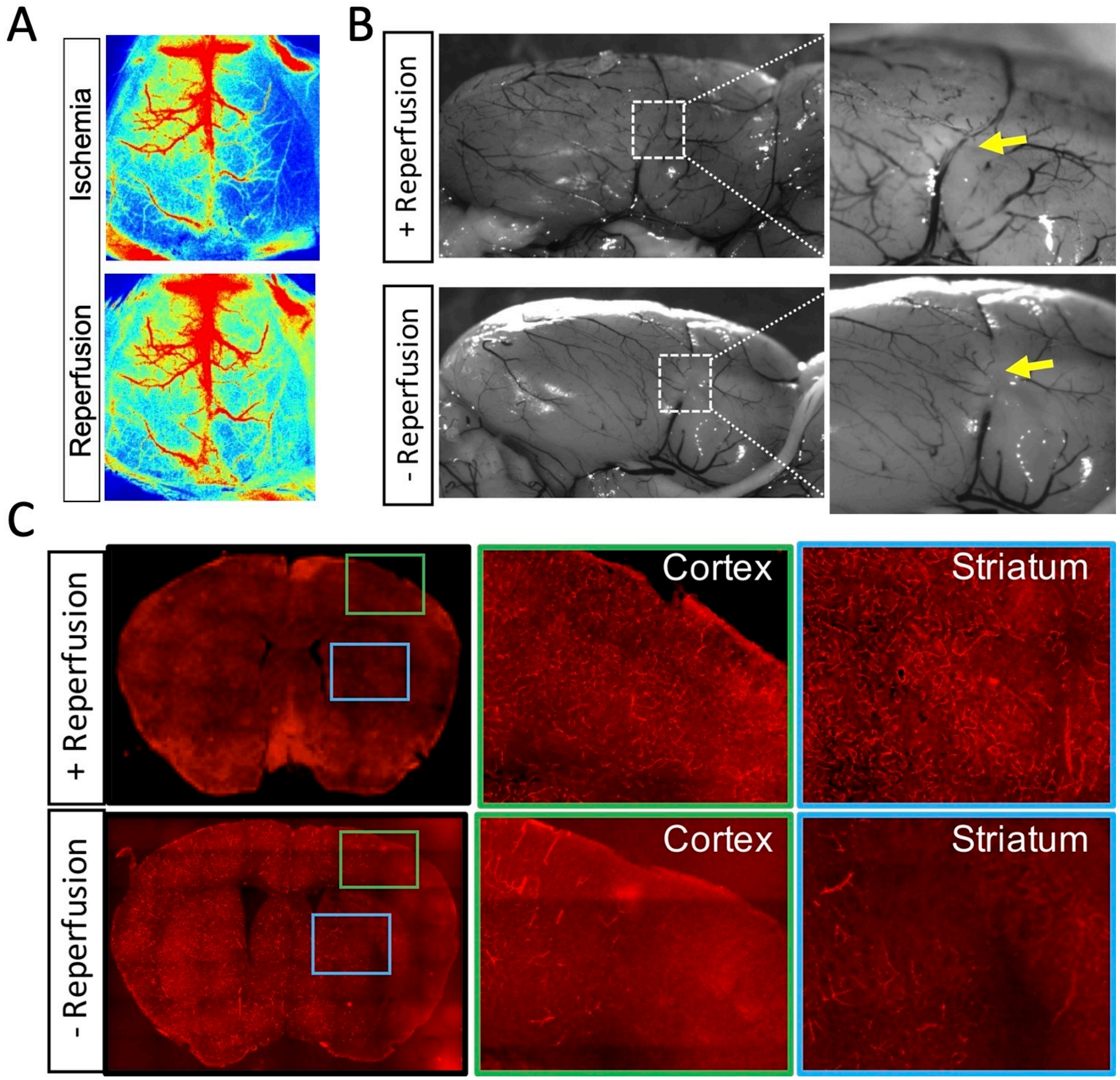

Figure S1. Evaluation of reperfusion after 6 hours ischemia in our ttMCAO model. A) Laser speckle contrast imaging (LSCI). Mice were subjected to occlusion of middle cerebral artery (MCA) at the site indicated in Fig. 1A. After 5 hours and 40 minutes MCAO, mice were anesthetized again, and the cortex was imaged by LSCI to show cerebral blood flow (CBF) during ischemia (top). Then, at 6 hours ischemia, both sutures from CCA and MCA were removed to initiate prefusion. Ten minutes later, CBF flux was imaged again using LSCI (bottom). **B)** Ink-perfused brain vessels. After 6 hours ttMCAO, mice were subjected to either reperfusion (+) or no (-) reperfusion. The mice were then transcardially

perfused with gelatin-India ink solution under controlled pressure. The right MCA ligated (yellow arrow) in this model was indicated in a representative image. **C)** Lectin labeling. After 6 hours ttMCAO, mice were subjected to either reperfusion (+) or no (-) reperfusion. Then, lectin DyLight 594 was injected through the external jugular vein. After 15 minutes, mice were perfused and fixed. Brains were sectioned, and the perfused brain vessels were visualized under a fluorescent microscope.

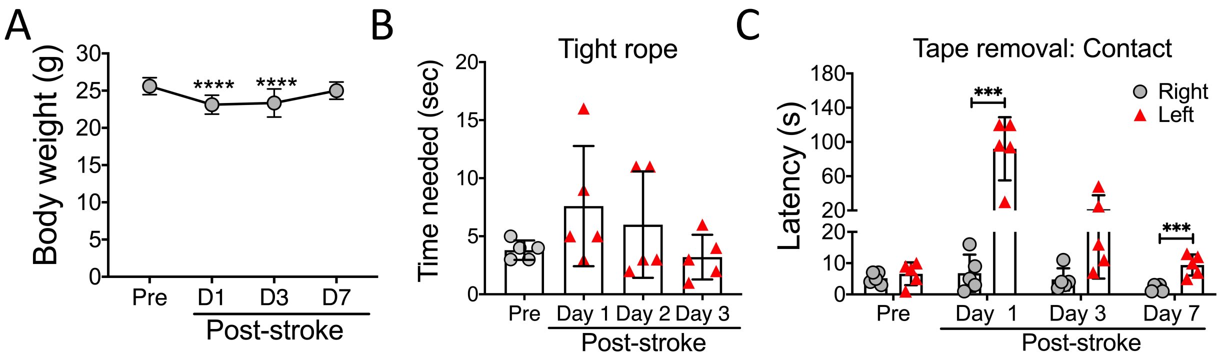

Figure S2. Stroke outcome after 6 hours ttMCAO in young mice. Supplemental information to Figure 1. Young C57Bl/6 mice were subjected to 6 hours ttMCAO. Data before stroke (Pre) are shown for comparisons. **A)** Body weight change in stroke mice. **B)** Tight rope test. **C)** Tape removal test. Data are presented as mean ± SEM (n = 5/group). *** $p < 0.001$; **** $p < 0.0001$.

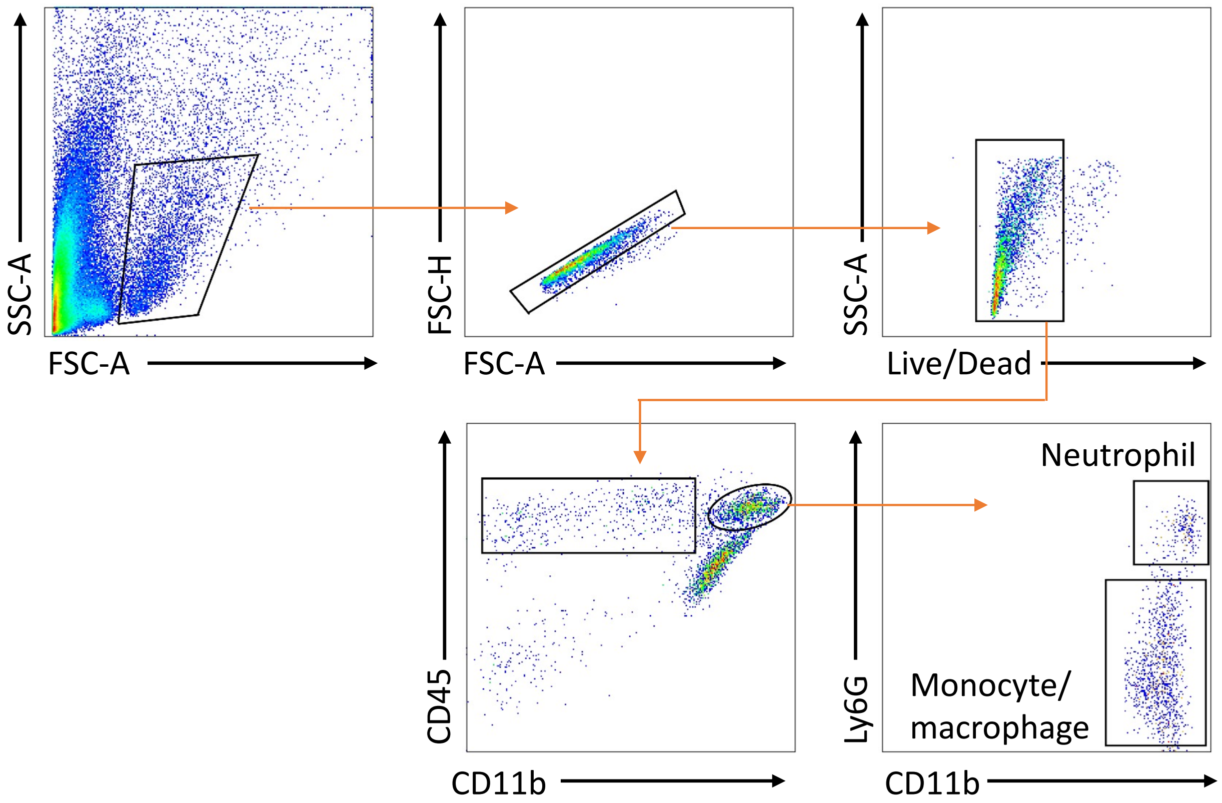

Figure S3. Flow cytometric gating strategy used in this study. Plots depict gating of infiltrated myeloid cells ($CD45^{hi}CD11b^{+}$), neutrophils ($CD45^{hi}CD11b^{+}Ly6G^{+}$), and monocytes/macrophages ($CD45^{hi}CD11b^{+}Ly6G^{-}$).

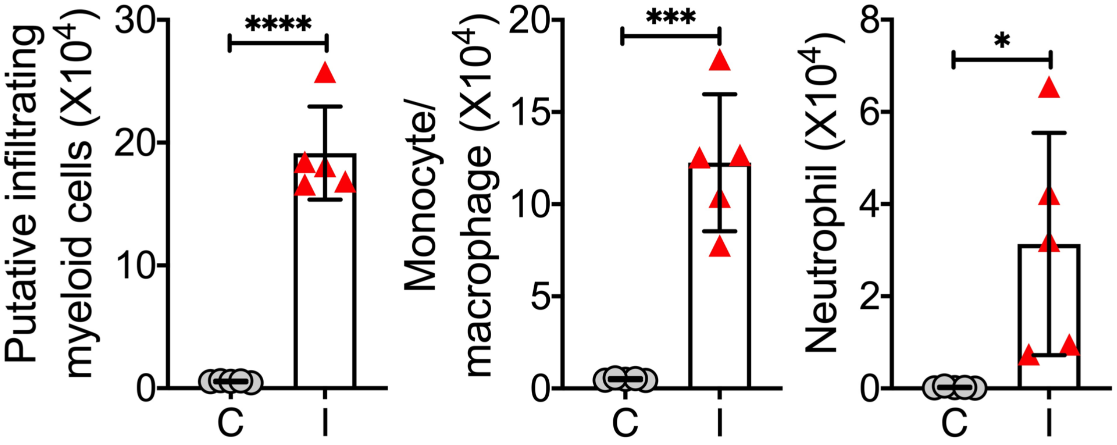

Figure S4. The flow cytometric analysis of immune cell populations in the aged brain after stroke. Aged mice (n = 5) were subjected to 6-hour ttMCAO. On day 3 after stroke, the contralateral (C) and ipsilateral (I) hemispheres were collected for flow cytometric analysis. The gating strategy is shown in Figure S3. Data are presented as mean ± SEM (n = 5/group). * $p < 0.05$; *** $p < 0.001$; ****$p < 0.0001$.

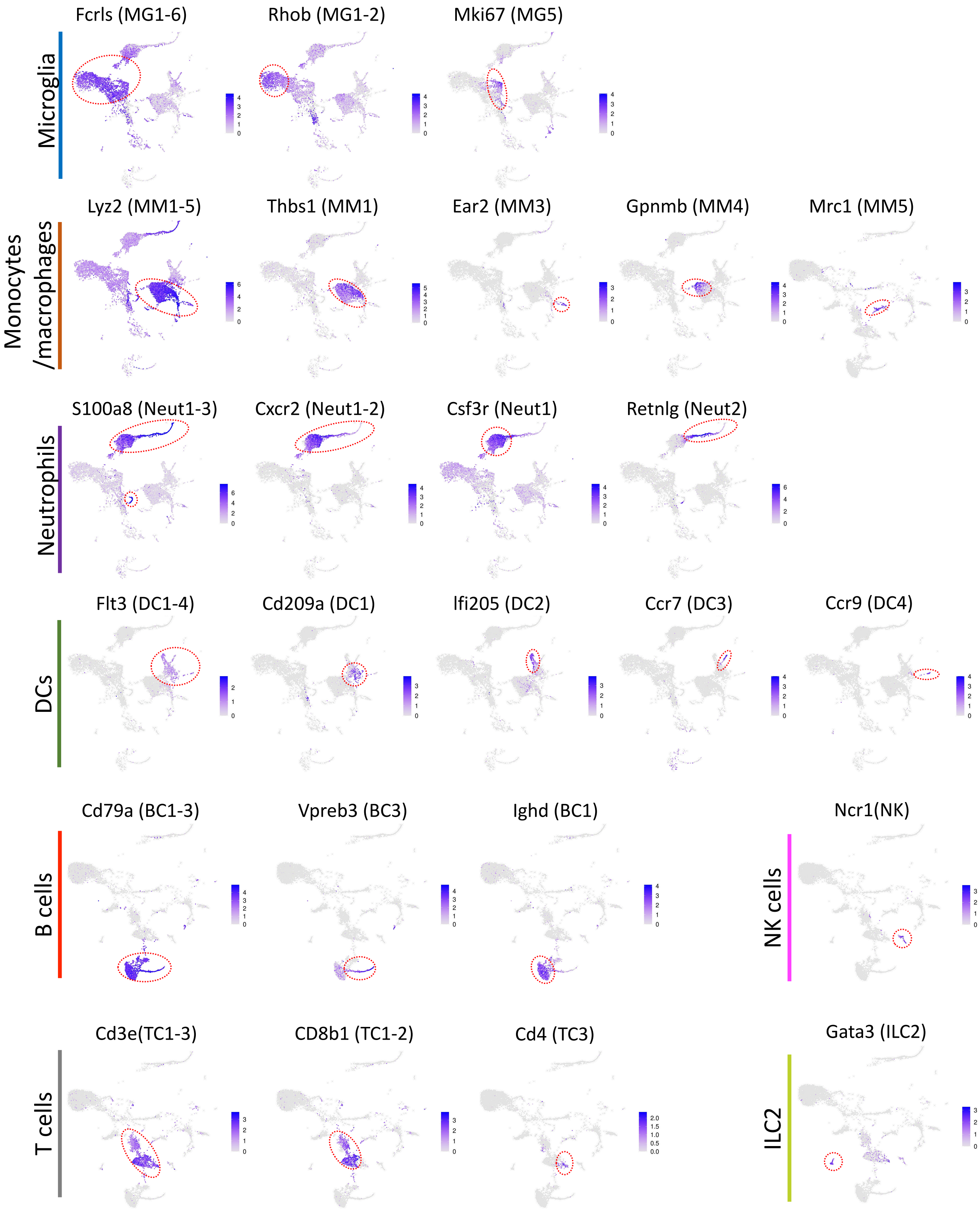
Microglia
Fcrls (MG1-6)
Rhob (MG1-2)
Mki67 (MG5)
Monocytes /macrophages
Lyz2 (MM1-5)
Thbs1 (MM1)
Ear2 (MM3)
Gpnmb (MM4)
Mrc1 (MM5)
Neutrophils
S100a8 (Neut1-3)
Cxcr2 (Neut1-2)
Csf3r (Neut1)
Retnlg (Neut2)
DCs
Flt3 (DC1-4)
Cd209a (DC1)
Ifi205 (DC2)
Ccr7 (DC3)
Ccr9 (DC4)
B cells
Cd79a (BC1-3)
Vpreb3 (BC3)
Ighd (BC1)
NK cells
Ncr1(NK)
T cells
Cd3e(TC1-3)
CD8b1 (TC1-2)
Cd4 (TC3)
ILC2
Gata3 (ILC2)

Figure S5. Molecularly distinct subpopulations of immune cells in the post-stroke aged brain. Supplemental information to Figure 2. Aged mice were subjected to 6-hour ttMCAO or sham. Three days later, both hemispheres (sham) or the ipsilesional hemisphere (stroke) were collected for scRNA-seq analysis of all FACS-isolated $CD45^+$ cells. Potential molecular markers for immune cell types or individual clusters were identified, and their expression was depicted on UMAP plots. To better visualize different cell populations, we plotted microglial, monocyte/macrophage, neutrophil, and DC populations in the stroke brain, and T-cell, B-cell, NK, and ILC2 populations in the sham brain. The color intensity indicates the expression level of each gene in individual cells. The main cell clusters for each gene are circled in red dotted lines.

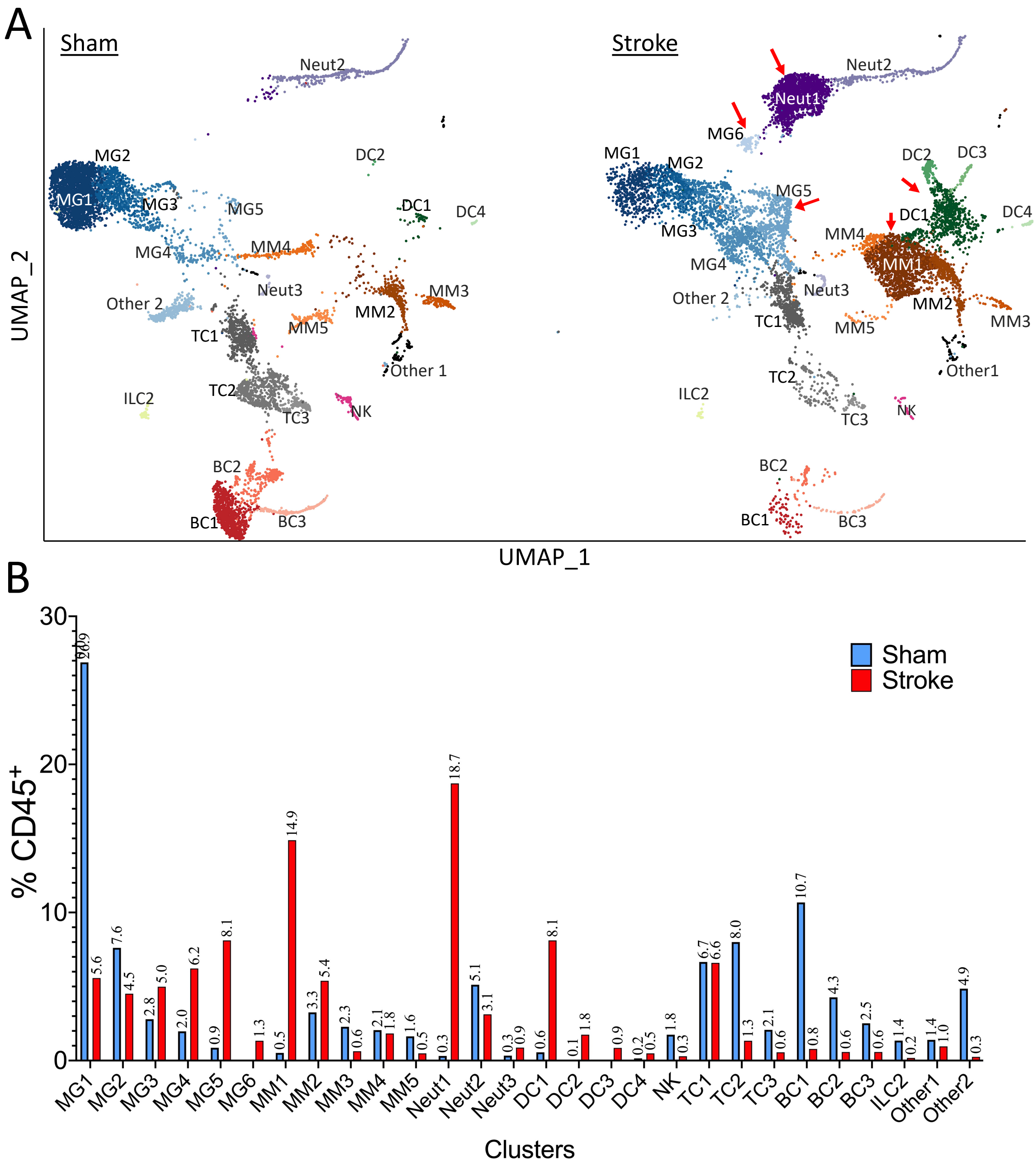

Figure S6. Changes of individual cell clusters in the aged brain on day 3 after stroke vs sham. Supplemental information to Figure 3. **A)** UMAP plots of cells from sham and stroke aged brains. The clusters with a marked increase in cell frequency after stroke (MG5, MG6, MM1, Neut1, and DC1) are indicated by red arrows. **B)** Changes in the frequency of each cell cluster after stroke vs sham.

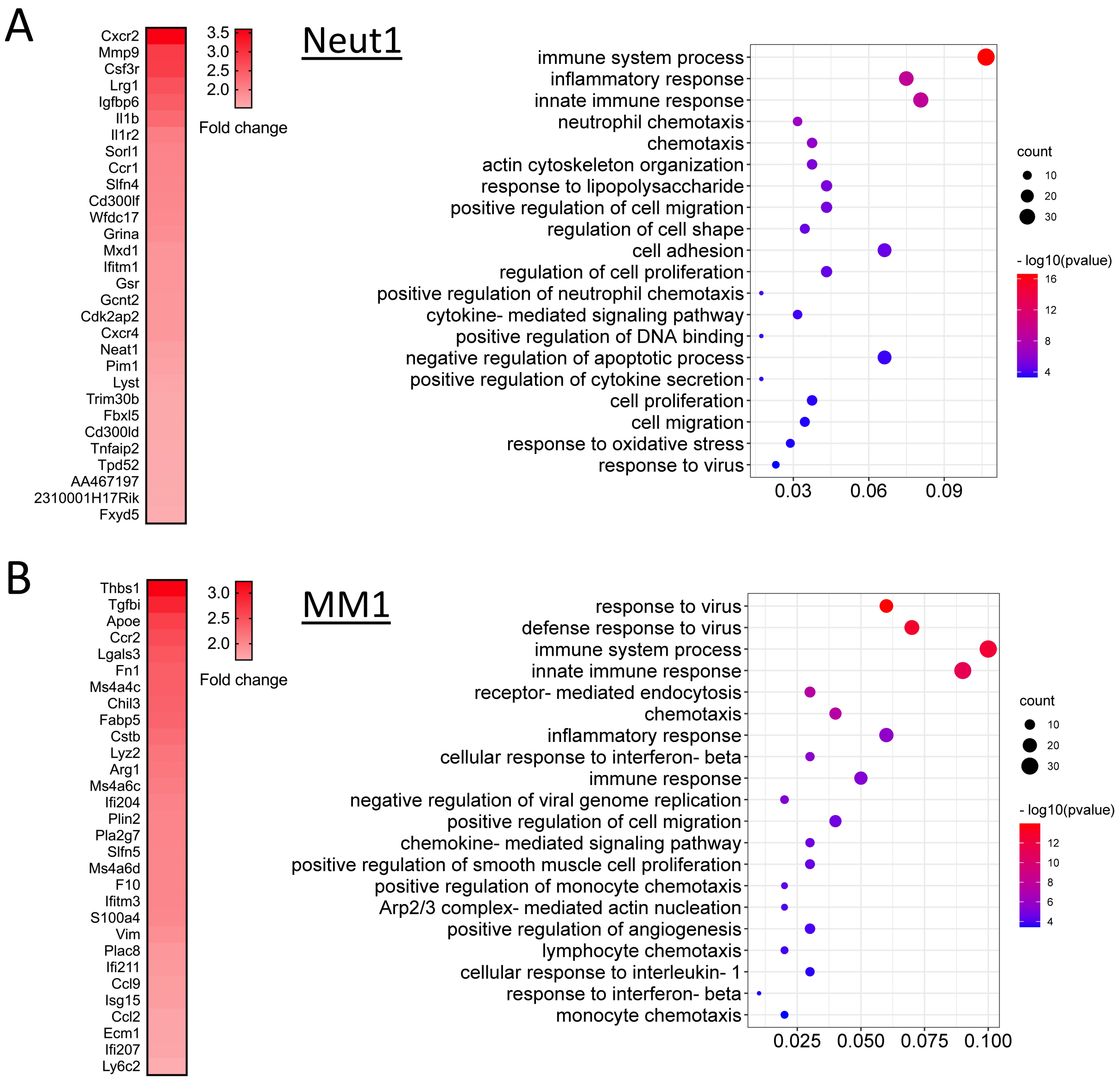
A
Cxcr2
Mmp9
Csf3r
Lrg1
Igfbp6
Il1b
Il1r2
Sorl1
Ccr1
Slfn4
Cd300lf
Wfdc17
Grina
Mxd1
Ifitm1
Gsr
Gcnt2
Cdk2ap2
Cxcr4
Neat1
Pim1
Lyst
Trim30b
Fbxl5
Cd300ld
Tnfaip2
Tpd52
AA467197
2310001H17Rik
Fxyd5
3.5
3.0
2.5
2.0
Fold change
Neut1
immune system process
inflammatory response
innate immune response
neutrophil chemotaxis
chemotaxis
actin cytoskeleton organization
response to lipopolysaccharide
positive regulation of cell migration
regulation of cell shape
cell adhesion
regulation of cell proliferation
positive regulation of neutrophil chemotaxis
cytokine- mediated signaling pathway
positive regulation of DNA binding
negative regulation of apoptotic process
positive regulation of cytokine secretion
cell proliferation
cell migration
response to oxidative stress
response to virus
0.03
0.06
0.09
count
10
20
30
- log10(pvalue)
16
12
8
4
B
Thbs1
Tgfbi
Apoe
Ccr2
Lgals3
Fn1
Ms4a4c
Chil3
Fabp5
Cstb
Lyz2
Arg1
Ms4a6c
Ifi204
Plin2
Pla2g7
Slfn5
Ms4a6d
F10
Ifitm3
S100a4
Vim
Plac8
Ifi211
Ccl9
Isg15
Ccl2
Ecm1
Ifi207
Ly6c2
3.0
2.5
2.0
Fold change
MM1
response to virus
defense response to virus
immune system process
innate immune response
receptor- mediated endocytosis
chemotaxis
inflammatory response
cellular response to interferon- beta
immune response
negative regulation of viral genome replication
positive regulation of cell migration
chemokine- mediated signaling pathway
positive regulation of smooth muscle cell proliferation
positive regulation of monocyte chemotaxis
Arp2/3 complex- mediated actin nucleation
positive regulation of angiogenesis
lymphocyte chemotaxis
cellular response to interleukin- 1
response to interferon- beta
monocyte chemotaxis
0.025
0.050
0.075
0.100
count
10
20
30
- log10(pvalue)
12
10
8
6
4

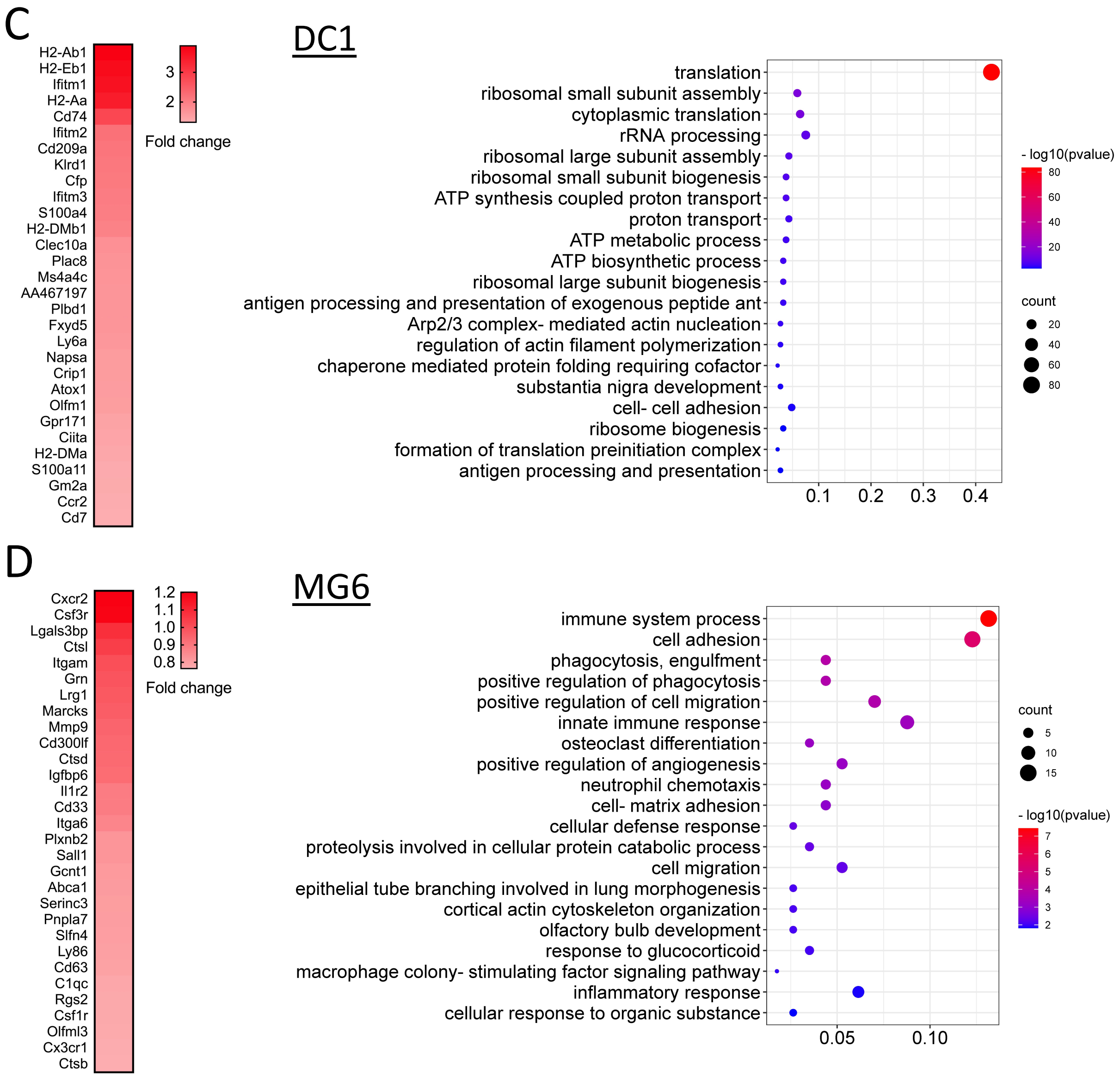

Figure S7. Major clusters with increased percentages after stroke. Four major clusters MM1 (A), Neurt1 (B), DC1 (C), and MG6 (D) are analyzed with DAVID program. Top 30 differentially expressed genes (DEGs) in each cluster are shown on the left. The fold change was calculated by comparing average expression of a gene in each cluster relative to the average expression of this gene in all other clusters combined. Cluster-enriched DEGs with *padj* < 0.01 and fold change >= 1.5 were subjected to gene ontology analysis. The top 20 overrepresented terms of biologic process are shown here (*right*).

References

1. Jiang M, Yu S, Yu Z, Sheng H, Li Y, Liu S, et al. Xbp1 (x-box-binding protein-1)-dependent o-glcnacylation is neuroprotective in ischemic stroke in young mice and its impairment in aged mice is rescued by thiamet-g. *Stroke*. 2017;48:1646-1654
2. Wang W, Li R, Miao W, Evans C, Lu L, Lyu J, et al. Development and evaluation of a novel mouse model of asphyxial cardiac arrest revealed severely impaired lymphopoiesis after resuscitation. *J Am Heart Assoc*. 2021;10:e019142
3. Parra A, McGirt MJ, Sheng H, Laskowitz DT, Pearlstein RD, Warner DS. Mouse model of subarachnoid hemorrhage associated cerebral vasospasm: Methodological analysis. *Neurol Res*. 2002;24:510-516
4. Wang Z, Li X, Spasojevic I, Lu L, Shen Y, Qu X, et al. Increasing o-glcnacylation is neuroprotective in young and aged brains after ischemic stroke. *Exp Neurol*. 2021:113646
5. Wang YC, Li X, Shen Y, Lyu J, Sheng H, Paschen W, et al. Perk (protein kinase rna-like er kinase) branch of the unfolded protein response confers neuroprotection in ischemic stroke by suppressing protein synthesis. *Stroke*. 2020;51:1570-1577
6. Taninishi H, Pearlstein M, Sheng H, Izutsu M, Chaparro RE, Goldstein LB, et al. Video training and certification program improves reliability of postischemic neurologic deficit measurement in the rat. *J Cereb Blood Flow Metab*. 2016;36:2203-2210
7. Jiang M, Li R, Lyu J, Li X, Wang W, Wang Z, et al. Mcc950, a selective nlpr3 inflammasome inhibitor, improves neurologic function and survival after cardiac arrest and resuscitation. *J Neuroinflammation*. 2020;17:256
8. Hammond TR, Dufort C, Dissing-Olesen L, Giera S, Young A, Wysoker A, et al. Single-cell rna sequencing of microglia throughout the mouse lifespan and in the injured brain reveals complex cell-state changes. *Immunity*. 2019;50:253-271 e256
9. McGinnis CS, Murrow LM, Gartner ZJ. Doubletfinder: Doublet detection in single-cell rna sequencing data using artificial nearest neighbors. *Cell Syst*. 2019;8:329-+
10. Macosko EZ, Basu A, Satija R, Nemesh J, Shekhar K, Goldman M, et al. Highly parallel genome-wide expression profiling of individual cells using nanoliter droplets. *Cell*. 2015;161:1202-1214
11. Satija R. Seurat-guided clustering tutorial 2017
12. van der Maarten L, Hinton G. Visualizing data using t-sne. *Journal of Machine Learning Research*. 2008;9:2579-2605
13. Tim Stuart AB, Paul Hoffman, Christoph Hafemeister, Efthymia Papalexi, William M. Mauck III, Marlon Stoeckius, Peter Smibert, Rahul Satija
